# USING MICRO-CREDENTIAL TO ADVANCE LONG-TERM CARE STAFF EXPERTISE IN PALLIATIVE CARE FOR PEOPLE WITH DEMENTIA

**DOI:** 10.1093/geroni/igae098.1133

**Published:** 2024-12-31

**Authors:** Winnie Sun, Jen Calver, Lucas Martignetti, Volletta Peters, Manon Lemonde

**Affiliations:** Ontario Tech University, Lindsay, Ontario, Canada; Ontario Tech University, Oshawa, Ontario, Canada; Ontario Tech University, OSHAWA, Ontario, Canada; Ontario Tech University, Oshawa, Ontario, Canada; Ontario Tech University, Oshawa, Ontario, Canada

## Abstract

**Background:**

Ontario Tech University partnered with experts from Alzheimer’s Society of Canada (Durham Region of Ontario) and Ontario Shores Centre for Mental Health Sciences to develop a micro-credentialing certificate program in dementia care. This study aims to evaluate the usability and applicability of using micro-credential learning to advance long-term care staff’s expertise in providing palliative care for people with dementia.

**Methods:**

Registered Practical Nurses (RPNs), Registered Nurses (RNs), Nurse Practitioners (NP) and Personal Support Workers (PSWs) (n=19) who worked in long-term care homes located in Southern Ontario completed a one-day training to pilot-test a gamified micro-credential education related to palliative care for people with dementia. Upon completion of the training, a quantitative pre-assessment and post-assessment were used to evaluate learner’s perceived knowledge, skill, attitude and confidence as measured by the Educational Self-Efficacy Scale.

**Results:**

Overall, there was a statistically significant increase in knowledge after the micro-credential training (P<.0001), with a mean increase of 3.2 more questions answered correctly. Focus group findings revealed four key themes: opportunities of using gamified simulation to promote learner’s engagement; lack of palliative care training to support people with dementia; need to strengthen workplace training using micro-credential for upskilling; and meaning of palliative care in long-term care sector.

**Conclusion:**

Palliative care is recognized to be an important part of work in long-term care as residents are often admitted with complex and life-threatening conditions. Participants highlighted the feasibility of enhancing their knowledge and competence in palliative care through specialized training using gamified, simulation learning through micro-credential training.

